# Exploring prognostic indicators in the pathological images of ovarian cancer based on a deep survival network

**DOI:** 10.3389/fgene.2022.1069673

**Published:** 2023-01-04

**Authors:** Meixuan Wu, Chengguang Zhu, Jiani Yang, Shanshan Cheng, Xiaokang Yang, Sijia Gu, Shilin Xu, Yongsong Wu, Wei Shen, Shan Huang, Yu Wang

**Affiliations:** ^1^ Department of Obstetrics and Gynecology, Shanghai First Maternity and Infant Hospital, School of Medicine, Tongji University, Shanghai, China; ^2^ Department of Obstetrics and Gynecology, Renji Hospital, School of Medicine, Shanghai Jiaotong University, Shanghai, China; ^3^ MoE Key Lab of Artificial Intelligence, AI Institute, Shanghai Jiao Tong University, Shanghai, China

**Keywords:** ovarian cancer, deep learning, prognosis, risk stratification, pathology

## Abstract

**Background:** Tumor pathology can assess patient prognosis based on a morphological deviation of tumor tissue from normal. Digitizing whole slide images (WSIs) of tissue enables the use of deep learning (DL) techniques in pathology, which may shed light on prognostic indicators of cancers, and avoid biases introduced by human experience.

**Purpose:** We aim to explore new prognostic indicators of ovarian cancer (OC) patients using the DL framework on WSIs, and provide a valuable approach for OC risk stratification.

**Methods:** We obtained the TCGA-OV dataset from the NIH Genomic Data Commons Data Portal database. The preprocessing of the dataset was comprised of three stages: 1) The WSIs and corresponding clinical data were paired and filtered based on a unique patient ID; 2) a weakly-supervised CLAM WSI-analysis tool was exploited to segment regions of interest; 3) the pre-trained model ResNet50 on ImageNet was employed to extract feature tensors. We proposed an attention-based network to predict a hazard score for each case. Furthermore, all cases were divided into a high-risk score group and a low-risk one according to the median as the threshold value. The multi-omics data of OC patients were used to assess the potential applications of the risk score. Finally, a nomogram based on risk scores and age features was established.

**Results:** A total of 90 WSIs were processed, extracted, and fed into the attention-based network. The mean value of the resulting C-index was 0.5789 (0.5096–0.6053), and the resulting *p*-value was 0.00845. Moreover, the risk score showed a better prediction ability in the HRD + subgroup.

**Conclusion:** Our deep learning framework is a promising method for searching WSIs, and providing a valuable clinical means for prognosis.

## 1 Introduction

Ovarian cancer (OC), as the “silent killer” of women’s health, is the leading cause of cancer-related death in gynecologic malignant diseases (Kuroki and Guntupalli, 2020). OC is a highly heterogeneous disease with a variety of subtypes that have various histologic and molecular characteristics (Kurman and Shih Ie, 2016), which raises challenges for effective prognosis stratification and clinical treatment management. High-throughput sequencing technologies have expedited research in cancer biology and provided a comprehensive genetic landscape (Lu et al., 2019). In recent years, more potential biomarkers for diagnosis and prognosis have been discovered based on the rapid advances in sequencing technologies. Similar to high throughput sequencing, the analysis of digital pathological images has provided an opportunity for biomarker detection and prognostic stratification (Desbois et al., 2020; Saillard et al., 2020; Skrede et al., 2020; Shi J. et al., 2021; Jin et al., 2021). Pathological analysis of OC patients is essential for obtaining patient diagnosis and cancer characteristics including histological subtype, grade and stage. Whole slide images (WSIs) harbor vast amount of information, such as growth patterns and intercellular interactions within tumor microenvironment, which is associated with the survival outcome. However, the high-dimensional information of pathology images cannot be recognized by the naked eyes of a pathologist.

Deep learning has presented outstanding advantages in medical image analysis due to its powerful feature representation (Litjens et al., 2017). Recent articles have shown that deep learning can enhance the analysis of pathology images for diagnostic and prognostic stratification (Skrede et al., 2020; Shi J. et al., 2021; Zhang X. et al., 2022). In practice, the labeling task mostly needs to spend much manual labor with experienced experts to implement a specific task for determining the target tissue. Especially, it is extremely challenging to finish a pixel-level labeling task for gigapixel images. Fortunately, the weakly-supervised learning approach can be exploited to alleviate this question because the clinical information almost includes a patient-level label (Lu et al., 2021).

Some works have investigated survival analysis based on time-to-event data *via* deep learning methods. Both learning the underlying dynamics of the modeling survival data and censoring are two important issues in the survival analysis. The right-censored cases led to the bias in the cross-Entropy-based model. Aimed at this question, the bias between them was analyzed systematically *via* different deep-learning model comparisons (Zadeh and Schmid, 2021). For right-censored data, the recurrent neural network was also utilized to conduct survival prediction and analysis. In addition, the survival loss function was also improved to reduce the bias by introducing the weight coefficient (Ren et al., 2019). Based on these works, multi-modality data was exploited and fused to predict the risk stratification for multiple types of cancers (Chen et al., 2022). However, risk stratification according to existing clinical indicators is not sufficient for OC patients. Thus, this study proposed a deep survival network based on WSIs to predict risk scores and obtained prediction of prognosis.

## 2 Materials and methods

### 2.1 Data collection and processing

For ovarian cancer prognostic analysis, we collected 106 patients’ H&E diagnostic WSIs with corresponding clinic data from TCGA-OV (https://portal.gdc.cancer.gov/projects/TCGA-OV) *via* the National Cancer Institute GDC Data Portal. The inclusion criteria herein consist of three aspects: 1) The quality of WSIs was assessed by an experienced clinic doctor; 2) retaining only one WSI for each case; 3) the case contained both clinical information and WSIs. As a result, 90 cases (60 uncensored patients and 30 censored ones) were incorporated to obtain a prediction model. Additionally, we exploited the five-cross validation method to train the model.

Herein, we utilized an open-source tool, CLAM WSI-analysis toolbox (Lu et al., 2021), to implement the segmentation and feature extraction tasks for each WSI. In this scenario, each original WSI consists of four levels of different resolutions. First, we segmented the tissue region of interest from the level 0 with the highest resolution in each WSI. Second, the whole slide image for each patient was split into M patches with 256 × 256 pixels without overlap. Third, a pre-trained deep network ResNet50 (trained on the ImageNet dataset) was exploited to extract feature tensors by feeding patches into it. Finally, the third block in the ResNet50 model was selected to output the feature tensor with 1,024 dimensions. Additionally, normalized gene expression was measured as Transcripts Per Kilobase Millions, and we processed the genetic mutation data of the TCGA dataset using the R package “maftools”.

### 2.2 Deep learning model

**FIGURE.1:**
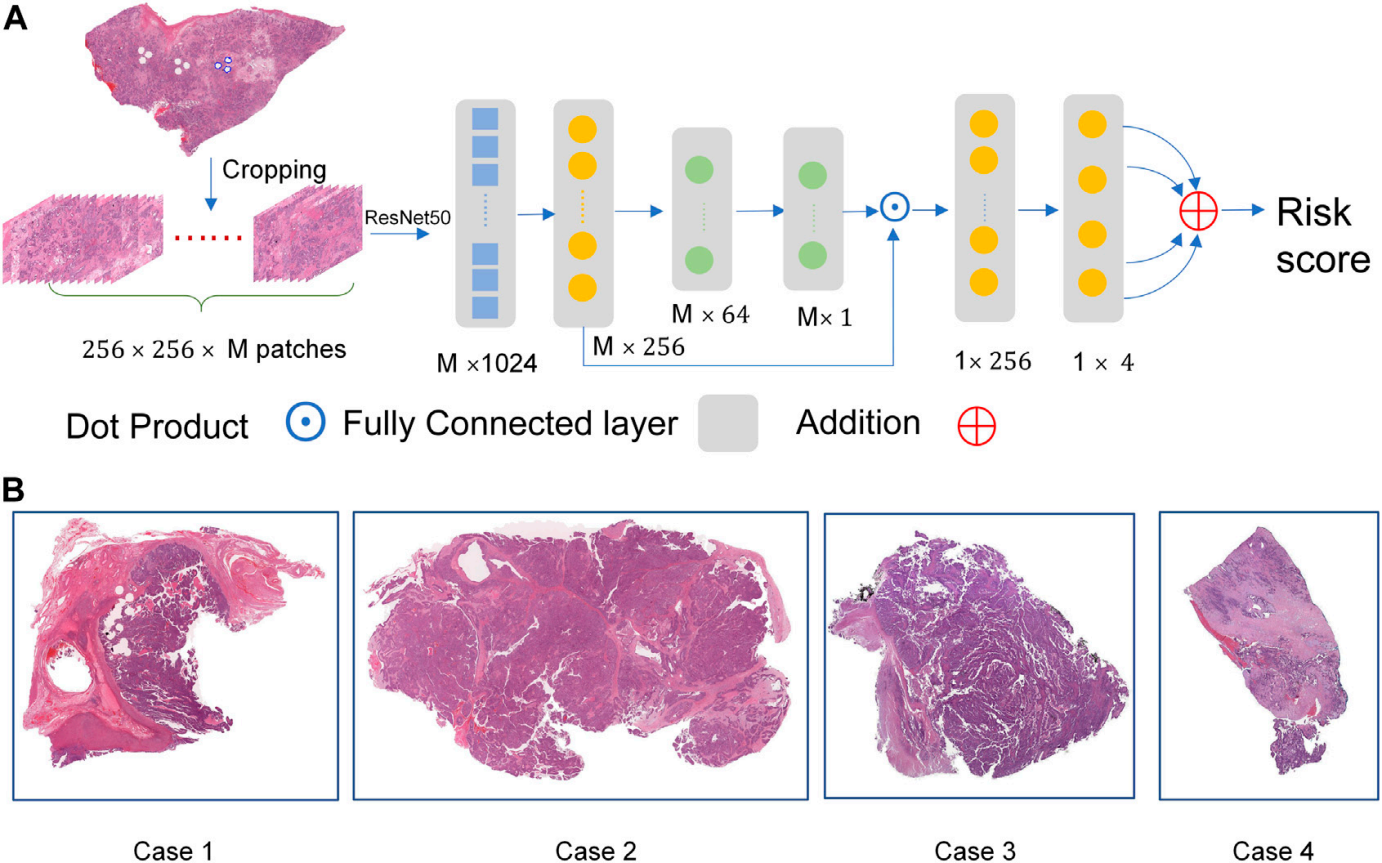
The overall network architecture. **(A)** The weakly-supervised deep learning architecture. **(B)** The representative images.

For each gigapixel WSI, it is a challenging task to provide pixel-level labels *via* human labor. The goal of survival data analysis is to train a predictor for hazard probability in a time interval based on plenty of image patches. Thus, we designed a weakly-supervised deep learning architecture as shown in Figure 1A, and the representative images were shown in Figure 1B.

We split the entire pipeline into two parts. In the first part, we extract M feature tensors X from M patches with 256×256 pixels *via* the pre-trained model. As we knew, the tissue region of interest varies with each WSI. In the second part, an attention-based network is designed to assign weights for each feature tensor as the input of the subsequent fully connected layer (FC). A set of learnable weight parameters are obtained for all patches feature for one case.

Due to the size of the dataset being slightly small, a two-layer network is designed to build the prediction layer. The overall network is optimized to predict the four hazard probabilities in four intervals *via* the loss function. The patient-level risk score is calculated by summing the four hazard scores. For subsequent analysis, the high-group and low-group are divided according to the median of all risk scores.

### 2.3 Loss function

To realize the survival analysis from patient-level data, we divide evenly the overall patient survival time into four intervals [*t*_*i*_,*t*_*i*+1_), *i* = 0,1,2,3 according to the uncensored cases. $Y_{j}\in \left\{0,1,2,3\right\}$ denotes the ground truth for the *j*th case. The subscript *j* denotes the *j*th patient. The prediction layer outputs the corresponding hazard probability as shown in Eq. 1.

$$f_{hazard}\left(r|x_{j}\right)=P\left(T_{j}=r|T_{j}\geq r,x_{j}\right)$$ (1)

where $x_{j}$ denotes the feature tensor for the *j*th case. Given $T_{j}\geq r$ and $x_{j}$ , the conditional probability $f_{hazard}\left(r|x_{j}\right)$ denotes the probability that the time period $T_{j}$ (in months) ends at time *r*. The survival function $f_{surv}$ is described in Eq. 2.

$$f_{surv}\left(r|x_{j}\right)=P\left(T_{j}>r|x_{j}\right)=\overset{r}{\underset{u=1}{\prod }}\left(1-f_{hazard}\left(u|x_{j}\right)\right)$$ (2)

The loss function is exploited as shown in Eq. 3 (Zadeh and Schmid, 2021).

$$\begin{array}{c} L=L_{censored}+L_{uncensored}\\ =-c_{j}\log\left(f_{surv}\left(Y_{j}|x_{j}\right)\right)-\left(1-c_{j}\right)\log\left(f_{surv}\left(Y_{j}-1|x_{j}\right)∙f_{hazard}\left(Y_{j}|x_{j}\right)\right) \end{array}$$ (3)

To balance the differences between the uncensored cases and censored counterparts, a hyper-parameter α is utilized in the final loss function ${\;L}_{surv}$ .

$$L_{surv}=\left(1-\alpha \right)L+\alpha L_{uncensored}$$ (4)

### 2.4 HRD analysis

The HRD score is calculated as the sum of telomeric allelic imbalance (TAI), large-scale state transitions (LST), and loss of heterozygosity (LOH) scores (Shi Z. et al., 2021). HRD scores are derived from research by Thorsson et al. (2018). HRD+ was defined as a high HRD score (threshold >42 score).

### 2.5 Tumor immune infiltration and pathway enrichment analysis

The relative infiltration level of immune cell types was quantified *via* single sample gene set enrichment analysis (ssGSEA) by the “GSVA” R package (Hanzelmann et al., 2013). GSEA was performed to assess related pathways.

### 2.6 Chemotherapeutic sensitivity prediction

Chemotherapeutic response prediction for OC samples was conducted in R by using the “oncoPredict” package (Maeser et al., 2021) from the Genomics of Drug Sensitivity in Cancer (GDSC) database (Yang et al., 2013). The ridge regression model was applied to evaluate the half maximal inhibitory concentration (IC50).

### 2.7 Nomogram construction

We performed a univariate analysis based on clinic parameters and risk scores. Afterward, multivariate Cox regression was conducted using the significant prognostic variables (*p* < 0.05). The nomogram was generated using the R package “rms”. We used calibration curves to test the consistency between predicted and actual survival rates. A time-dependent Receiver operating characteristic (ROC) curve was also used to assess the predictive accuracy of the nomogram. In addition, the Decision Curve Analysis (DCA) was used to demonstrate the advantage of the prediction curve using the R package “ggDCA”.

### 2.8 Statistical analysis

The statistical significance for variables with non-normal distribution was analyzed using the Wilcoxon rank sum test. The comparison between two groups of variables with normal distribution was estimated using an unpaired Student’s *t*-test. Non-parametric correlation analyses were conducted based on Spearman’s rank correlation coefficient. The prognostic analysis was performed by the Kaplan-Meier analysis method, and the log-rank test was used to evaluate significant differences. All statistical analyses were conducted using Python software (version 3.7) and R software (version 4.1.3). *p* < 0.05 was considered statistically significant.

## 3 Results

### 3.1 The training and validation of deep neural network

Overall 90 cases picked from the original TCGA-OV dataset were divided randomly into training (72 cases) and test (18 cases) datasets, respectively. We initialized the network parameters with “nn.init ()” and adopted the Adam solver with a momentum of 0.9 for the training process. Batch size, epoch number, and α are initialized as 1.40, 0.31, respectively. Additionally, the initial learning rate is set to 0.0002 and decayed with a Cosine Annealing schedule (Loshchilov and Hutter, 2017). To verify the effectiveness of the proposed network model proposed, we conducted a 5-fold cross-validation experiment on one NVIDIA GeForce RTX 3090 GPU. We utilize Pytorch based on Python 3.7 to implement all training and test tasks.

**FIGURE 2:**
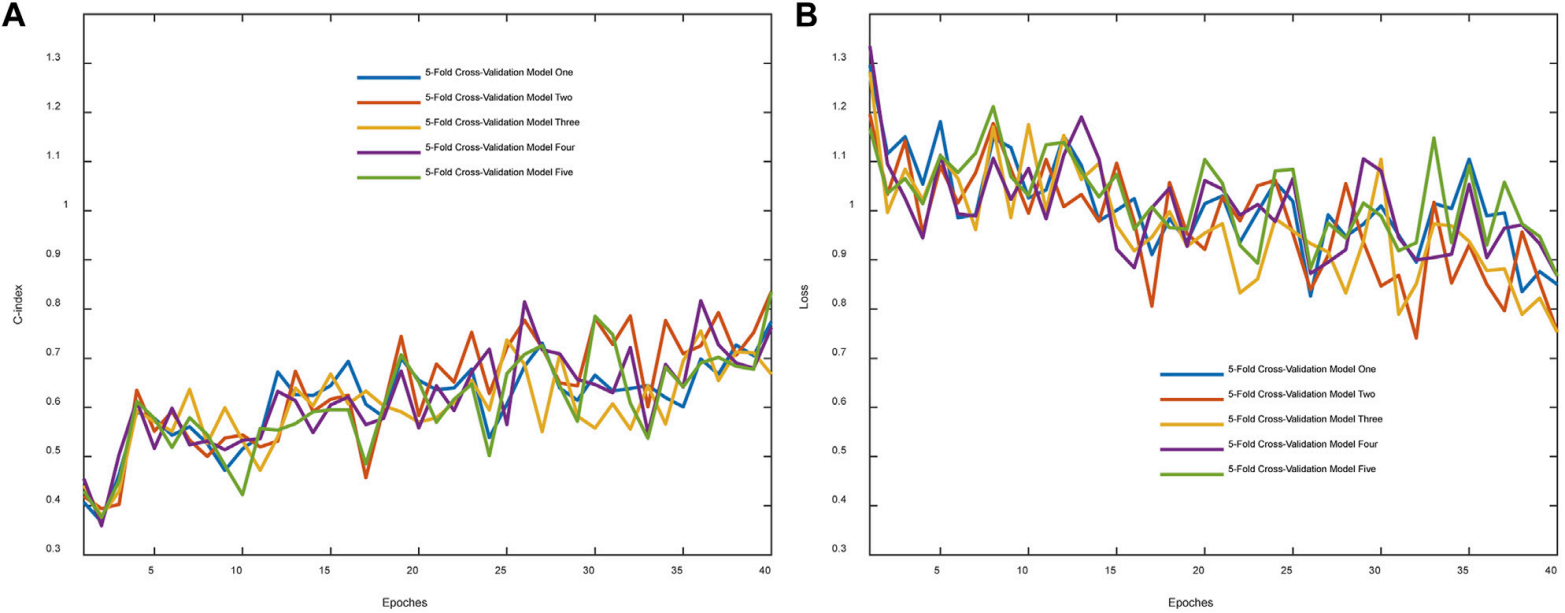
Evaluation metric and training loss were visualized. **(A)** The C-index changes with the epoch. **(B)** The whole loss reduces with the epoch.

C-index was originally proposed to evaluate predictions for binary responses (Harrell et al., 1996). As an evaluation metric utilized widely, it is herein utilized to evaluate the network performance. Figure 2A demonstrated that the C-index overall increased in the process of training. The C-index in the cross-validation experiment varies from 0.5096 to 0.6053 and the corresponding mean value was 0.5789. Figure 2B showed that the loss function reduces overall in 5-fold cross-validation results.

**FIGURE 3:**
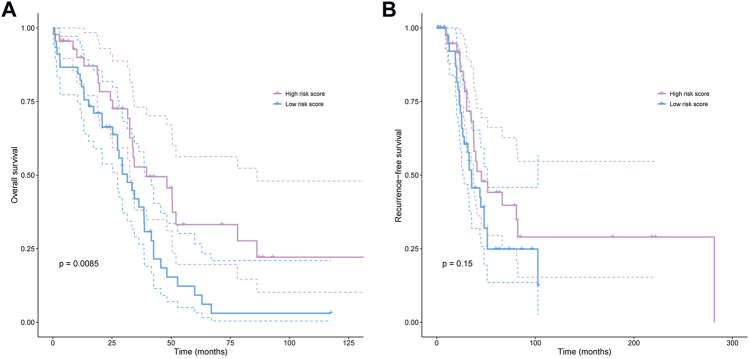
The Kaplan-Meier curve was plotted based on the prediction hazard value for each case. **(A,B)**, The overall [**(A)**, *p* = 0.0085] and recurrence-free [**(B)**, *p* = 0.15] survival difference between high- and low-risk score groups.

To verify further the effectiveness of the deep survival network, the KM curve was employed to analyze the prediction results. The high- and low-risk cohort was classified *via* the median value of the sorted hazard value associated with each patient. The KM cure of overall survival and recurrence-free survival were plotted as shown in Figures 3A, B. In addition, the *p*-value was calculated to analyze these two distributions based on the log-rank test. *p*-value of 0.00845 demonstrated that there was a significant difference between the high- and the low-risk score group The clinical characteristics between the high and low risk score subgroups, including age, stage, grade, and residuals were presented in Table1 to provide a clearer understanding of the sample distribution. Detailed risk score and clinical characteristics were shown in Supplementary Table S1.

**TABLE 1:** Characteristics between high- and low-risk score groups.

Characteristics	Overall (*N* = 90)	High risk score group (*N* = 45)	Low risk score group (*N* = 45)	*p*-value
Risk score (mean ± SD)	2.33 ± 0.42	2.66 ± 0.18	2.01 ± 0.33	<0.001
Age (mean ± SD)	59.96 ± 10.84	58.13 ± 10.40	61.78 ± 11.08	0.111
Stage (n, %)				0.494
I	2 (2.2)	0 (0.0)	2 (4.4)	
II	3 (3.3)	2 (4.4)	1 (2.2)	
III	62 (68.9)	32 (71.1)	30 (66.7)	
IV	22 (24.4)	11 (24.4)	11 (24.4)	
Unknow	1 (1.1)	0 (0.0)	1 (2.2)	
Grade (n, %)				0.234
G2	2 (2.2)	0 (0.0)	2 (4.4)	
G3	84 (93.3)	44 (97.8)	40 (88.9)	
Unkown	4 (4.4)	1 (2.2)	3 (6.7)	
Residual (n, %)				0.324
0	4 (4.4)	2 (4.4)	2 (4.4)	
1–10 mm	44 (48.9)	25 (55.6)	19 (42.2)	
11–20 mm	7 (7.8)	1 (2.2)	6 (13.3)	
>20 mm	15 (16.7)	8 (17.8)	7 (15.6)	
No macroscopic disease	20 (22.2)	9 (20.0)	11 (24.4)	

### 3.2 Predicting survival outcome of HRD patients *via* risk score

**FIGURE 4:**
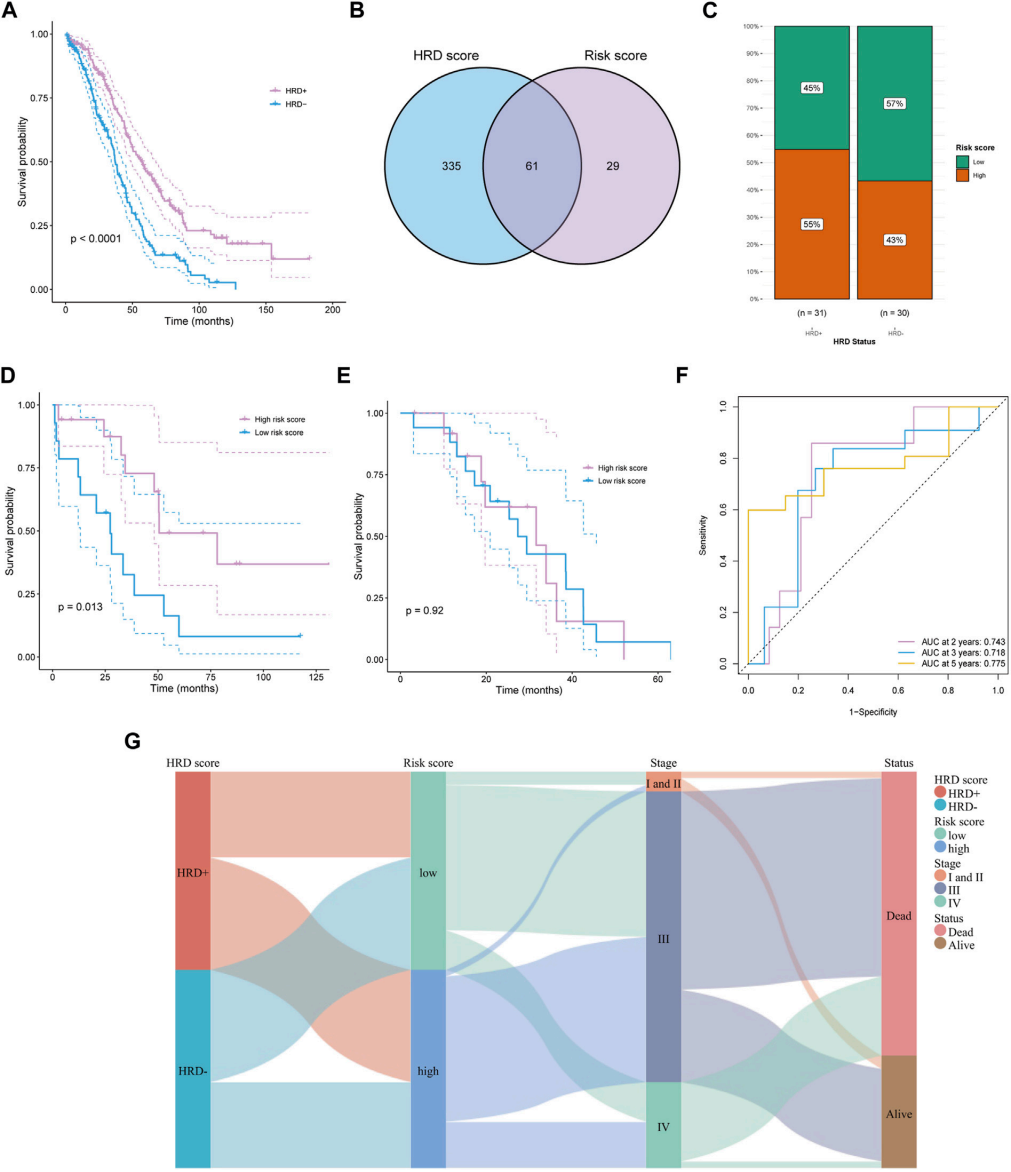
Predicting survival of HRD patients. **(A)** The survival difference between HRD+ and HRD-groups. **(B)** Obtaining HRD score and risk score intersections with venn diagrams. **(C)** In the HRD + subgroup, a high percentage of people with high risk score. **(D, E)** The survival difference in HRD + subgroup **(D)** and HRD-group **(E)** between high and low risk score groups. **(F)** Time dependent ROC curves of risk score in HRD + group at 2, 3, and 5 years. **(G)** Described the relationship among HRD score, risk score, stage, and survival status by sankey diagram.

Platinum-based chemotherapy is the essential treatment for OC. Patients with HRD+ (HRD score> 42) and BRCA1/2 mutation are more beneficial from chemotherapy, thus, we evaluated the prognostic applications of the risk score in HRD+ and HRD-subgroups. In the TCGA-OV cohort, the HRD + subgroup displayed a significantly better OS than HRD-group (*p* < 0.0001, Figure 4A). 61 overlapped patients with WSIs and HRD scores were detected (Figure 4B). The HRD + subgroup harbored mainly high-risk score population, while the HRD-group revealed an opposite result (Figure 4C), which suggested that the risk score may work in a different way from the method based on HRD in predicting survival outcomes. Moreover, survival analysis demonstrated a significantly better OS in patients with high-risk score than that with low-risk score in HRD + subgroup (*p* = 0.013; Figure 4D). Interestingly, there was no significant difference in HRD-group comparing the OS of the two risk populations (*p* = 0.92, Figure 4E). ROC curve analysis is used to assess the sensitivity and specificity of prediction models and validate the results of risk prediction values. The time-dependent ROC curve proved the reliable performance of risk score in HRD + subgroup (2-years AUC = 0.743, 3-years AUC = 0.718, 5-years AUC = 0.775, Figure 4F). To present the HRD score, FIGO staging, survival status, and risk score calculated by WSI as a unified system, Sankey diagram was constructed to describe the relationship between these features (Figure 4G).

### 3.3 Mutant landscape and immune infiltration

**FIGURE 5:**
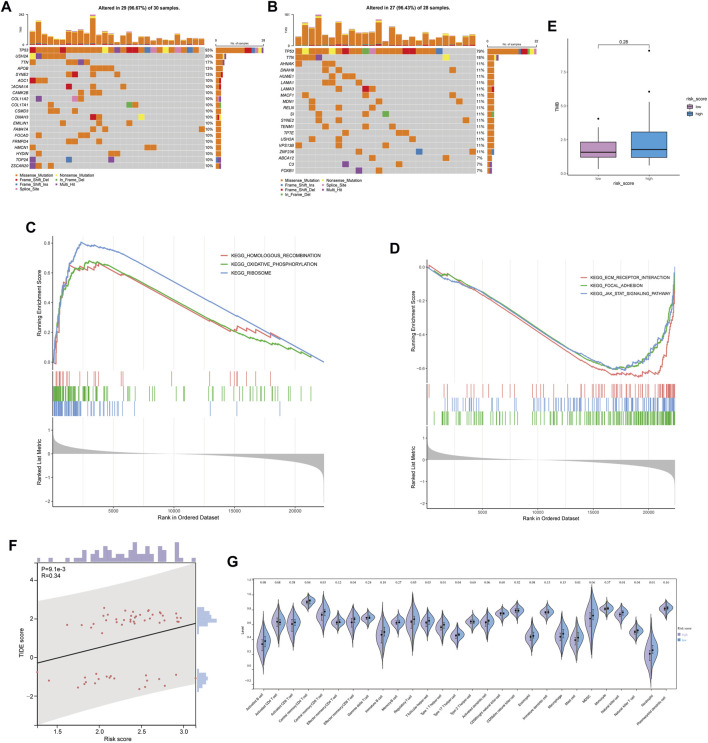
Mutant landscape and immune infiltration between high and low risk score groups. **(A,B)** Mutation landscape of OC patient with low risk score **(A)** and high risk score **(B)**. **(C,D)** Enrichment analysis based on GSEA in high risk score group **(C)** and low risk score group **(D)**. **(E)** Comparison of tumor mutation burden between high and low risk score groups. **(F)** Relationship between risk score and TIDE score. **(G)** Relationship between risk score and immune infiltration.

The relationship between risk score and mutation landscape has been assessed in OC patients. In both high- and low-risk score groups, the top 20 mutated genes were presented in Figures 5A,B. TP53 (93%), USH2A (20%), and TTN (17%) exhibited the most frequent mutations in the high-risk score group, while the highly mutant genes in the low-risk score group were TP53 (79%), TTN (18%) and AHNAK (11%). Moreover, the high-risk score group genes were enriched in HOMOLOGOUS RECOMBINATION, OXIDATIVE_PHOSPHORYLATION, and RIBOSOME pathway (Figure 5C), as well as the low-risk score group genes were enriched in FOCAL ADHESION, JAK STAT SIGNALING PATHWAY, and ECM RECEPTOR INTERACTION pathway (Figure 5D). The mutation (Supplementary Tables S2, S3) and GSEA data (Supplementary Table S4) were shown in Supplementary Material. We also tried to analyze the correlation between the risk score and TMB, but got a negative result (Figure 5E). Since the high antigenicity induced by tumor mutations can recruit a large number of immune cells, we studied the relationship between risk and TIDE score, to evaluate the predictive value of risk score in immunotherapy outcomes. Unfortunately, although the risk score was correlated with TIDE (*p* = 0.0091, Figure 5F), the spearman correlation coefficient was relatively low. These results were consistent with the poor response of ovarian cancer to immunotherapy. Next, we analyzed the relationship between risk score and immune cell infiltration by ssGSEA. Several types of immune cells, such as the central memory CD4 T-cell, central memory CD8 T-cell, and effector memory CD8 T-cell, were found significantly less recruited in high-risk group tumor environment (Figure 5G). The risk score may be a supplemented as an indicative tool to further investigate immune cell infiltration in ovarian cancer.

### 3.4 Chemotherapeutic response analysis

**FIGURE 6:**
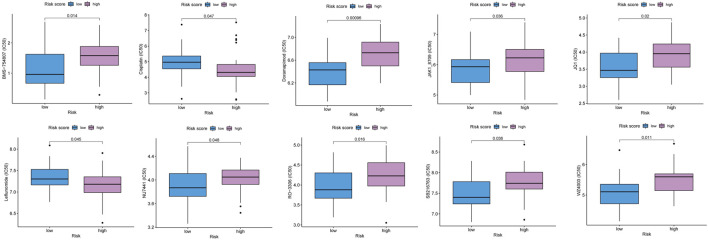
The predicted IC50 for chemotherapeutic drugs in the low and high risk score groups.

We attempted to identify whether the risk score could be applied to predict the sensitivity of response to chemotherapies using the GDSC database. The results revealed that the low-risk score group had a lower half maximal inhibitory concentration of BMS-754807, doramapimod, JAK1_8709, JQ1, NU7441, RO3306, SB216763, and WZ4003, while the high-risk score group had a lower half maximal inhibitory concentration of cisplatin, leflunomide (Figure 6). The p38MAPK inhibitors ralimetinib have been shown to play an anti-cancer role in ovarian cancer (Campbell et al., 2014). While Doramapimod also showed potent anti-inflammatory effects as p38MAPK inhibitors (Schreiber et al., 2006), perhaps our study will explore a new alternative for the application of Doramapimod in ovarian cancer. Similarly, RO3306 (Yang et al., 2016), SB216763 (Kaltofen et al., 2020) were also shown to play an anti-cancer role in ovarian cancer. Moreover, the combination of JQ1 and cisplatin helped ovarian cancer-bearing mice survive (Yokoyama et al., 2016). However, previous study showed that NU7441 could induce resistance to PARP inhibitor in BRCA1-defective cells (McCormick et al., 2017), and BMS-754807 combined with carboplatin/paclitaxel was observed resistance in ovarian carcinosarcoma of patient-derived xenograft (Glaser et al., 2015). Therefore, we should actively explore new strategies for more drug combinations to avoid the resistance in ovarian cancer. Our model provided possibility for novel pathways of drugs. Also, we hope that more pre-clinical models will prove our predicted results.

### 3.5 Development of a nomogram for predicting survival

**FIGURE 7:**
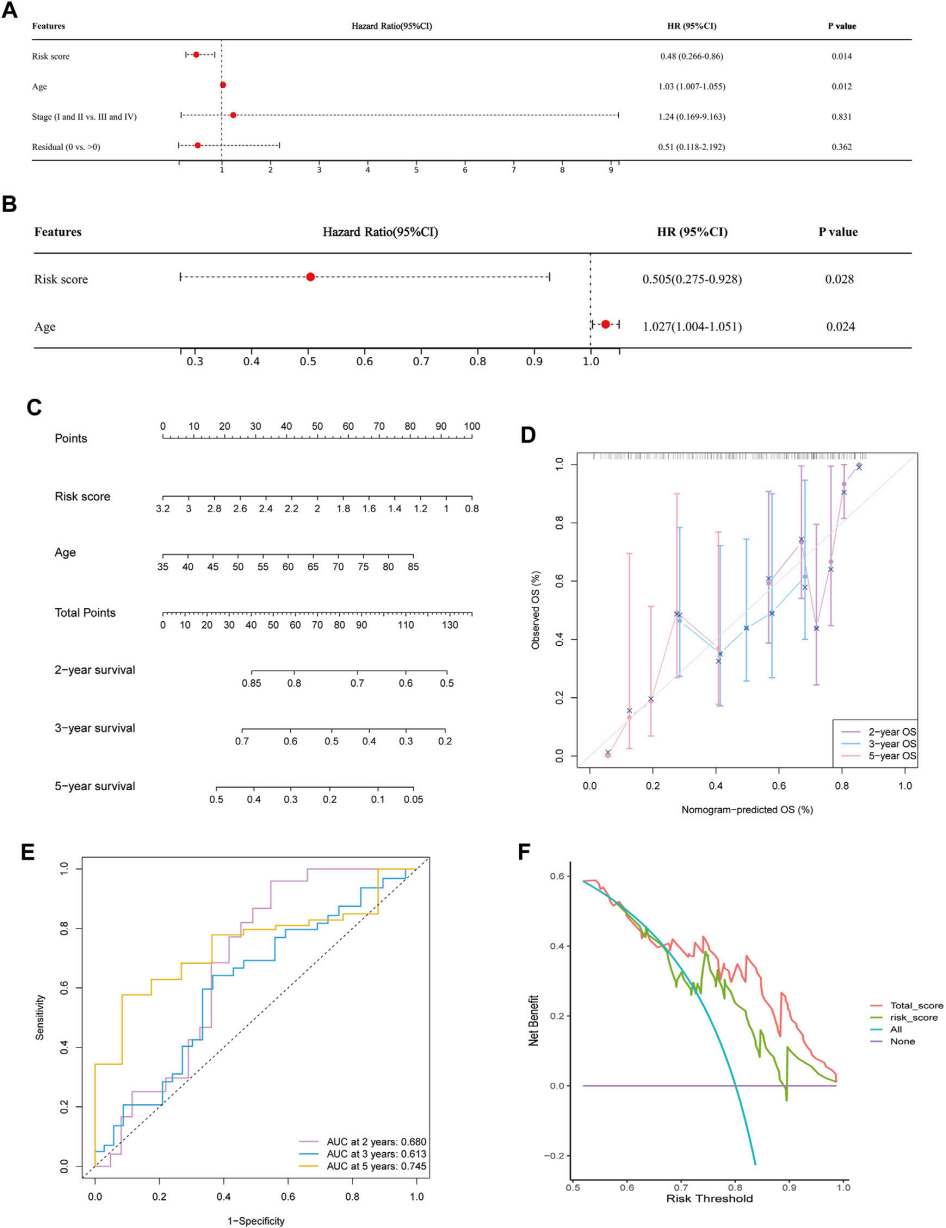
Predicting survival by integrating risk score and clinical feature. **(A,B)** Univariate **(A)** and multivariate Cox regression analysis **(B)** showed risk score and age were significantly correlated with overall survival. **(C)** Nomogram was constructed to predict the 2-, 3-, and 5-years survival of OC patients. **(D)** Calibration curve of the nomogram for predicting the probability of OS at 2, 3 and 5 years. **(E)** Time dependent ROC curves of nomogram at 2, 3 and 5 years. **(F)** Decision curve analysis of OS for the predicted nomogram model.

Based on the available clinic features, Cox regression analyses were conducted to identify the possibility that risk score was an independent prognostic factor for OS. The univariate Cox regression analysis revealed significant associations between risk score and OS (HR = 0.48, 95% CI = 0.266–0.86, *p* = 0.0138, Figure 7A). When other confounding factors were corrected, multivariate Cox regression analysis proved the risk score of WSIs was an independent predictor of prognosis (HR = 0.505, 95% CI = 0.275–0.928, *p* = 0.028, Figure 7B).

Following the results of the Cox regression analysis, we have further developed a nomogram incorporating two independent prognostic factors (risk score and age) to offer a quantitative method for estimating 2-, 3-, and 5-years survival rates of OC patients **(** Figure 7C). In addition, calibration plots showed that the nomogram was comparable to an ideal model **(** Figure 7D **).** At 2, 3, and 5 years, the AUC of the risk score was 0.605, 0.578, and 0.680. Nomogram’s accuracy of prediction over 2, 3, and 5 years was significantly higher, at 0.680, 0.613, and 0.745, respectively **(** Figure 7E **)**. DCA result also indicated our nomogram had a promising potential for clinical application **(** Figure 7F)**.**

## 4 Discussion

Tumor histology remains essential in predicting tumor aggressiveness and evaluating prognostic stratification. Previous studies have proved that deep learning models, such as DeepSurv (Katzman et al., 2018)and AECOX (Huang et al., 2020), can provide better predicted performance than traditional Cox regression model in predicting prognosis since learning the complex non-linear interactions (Tran et al., 2021). In addition, several recent studies have provided preliminary evidence that deep learning can help predict patient prognosis from digital pathology images (Skrede et al., 2020; Shi J. et al., 2021), but it is yet unclear how this contributes to OC risk categorization. In our study, we showed that WSIs data had a predictive capacity for survival.

Based on this concept, we exploited an attention-based network architecture to predict the hazard function based on patient-level labels. Unfortunately, the sample size of the TCGA-OV obtained 106 cases and only 90 cases were incorporated into this experiment finally. It was prone to overfitting for small sample datasets. To overcome the overfitting issue, we reduced the parameter number of FC. In the modeling process, the cross-entropy loss function and negative log-likelihood loss function are popular approaches. (Zadeh and Schmid (2021) analyzed the bias between two loss functions. Experiment results showed that the cross-entropy-based network tended to be similar to the respective results obtained from log-likelihood-based training if the censoring rate was high. The censored/uncensored case belongs to two kinds of survival data. For deep learning algorithms, we generally assume that the training dataset and test one are subject to the same distribution. Especially, The TCGA-OV dataset herein only obtained 90 cases after deleting some unsuitable cases. Thus, we randomly split the dataset and made the training have a similar portion in each fold. Of course, we conducted the same operation in the test dataset. The experiment results showed that the network presents a good performance in terms of the C-index. However, more cases incorporated will be helpful to enhance the model’s accuracy. The prognostic analysis of ovarian cancer by pathological images was included in the previous pan-cancer analysis, which achieved a c-index of 0.57243 (Fu et al., 2020), but our c-index was up to 0.6053. Moreover, our method automatically selects regions of interest (ROI) from the entire tissue field, which will allow pathologists to enhance standard clinical workflows without additional manual steps. And previous study has successfully predicted the recurrence in breast cancer without ROI label (Phan et al., 2021). We believe that the approach will avoid subjective bias.

We also performed analysis at the molecular level associated with the pathological images, including in HRD subgroup analysis, pathway enrichment analysis, etc., which was not seen in the previous study. OC patients with HRD can increase sensitivity to PARP inhibitors and improve overall survival benefits. Previous studies have shown that integrating image information and HRD status using machine learning methods has increased the capability of prognostic prediction for OC patients (Boehm et al., 2022), but no studies are using WSIs data to assess prognosis in HRD patients. Thus, we researched the survival differences in the HRD + subgroup with a risk score. The results showed that patient survival with a high-risk score was better than that with a low-risk score. And AUC of time-dependent ROC curves verified the reliable predicted performance of the risk score. Thus, the pathological images can not only determine the risk stratification of overall ovarian cancer patients but also differentiate the prognosis of HRD subgroups of patients, which may provide options for targeted therapy in OC patients.

Tumor microenvironment (TME) has been proved to play an essential role in tumor proliferation, migration and metastasis (Jiang et al., 2020). Here we found the low risk score group slides harbor complex cellular components as a common characteristics in WSI. In addition, Park et al. (2022) analyzed tumor-infiltrating lymphocytes based on AI using WSI, which illustrated the relationship between the characteristics of WSI and TME. The similar relationship between risk score based on WSI and immune cell infiltration was presented in our study.

Ovarian cancer with low tumor-infiltrating lymphocyte is considered “cold” tumor (Yang et al., 2022). The results of our immune infiltration analysis also showed no difference in activated CD8 T-cells between the high and low risk score subgroups. Interestingly, we found that there was significant difference in central memory T-cells, such as central memory CD8 and CD4 T-cells. The central memory T-cells have high proliferative potential compared to effector memory T-cells, which contributes to the formation of the patient’s immune memory pool (Sallusto et al., 2004). Sckisel et al. (2017) showed that the composition of the memory pool at different loci had a strong influence on the overall expression of those markers in the memory pool. For example, the memory ratio of CD8 subpopulations was more skewed toward central memory T-cell in the lymphoid region (Sallusto et al., 2004). Therefore, we hypothesized that the characteristic marker distribution of the immune memory pool in ovarian cancer patients could be detected by the advantage of spatial visualization of WSI, and predicting the recurrence and survival of patients. However, there are fewer studies on central memory T-cells in ovarian cancer, and experiments will be needed to explore in the future.

By GSEA analysis, we found that pathways associated with HRD were enriched, such as homologous recombination pathways. DNA can be repaired by high-fidelity homologous recombination when double-stranded damage occurs. The risk of cancer will increase when HR is dysregulated (Helleday, 2010). In addition, we observed that the ribosomal pathway was also enriched. It has been shown that deficient ribosome assembly was associated with cancer, while mutation in ribosomal proteins regulated the translation and activity of p53, ultimately leading to disease (Goudarzi and Lindstrom, 2016). Interestingly, it was found that pathways associated with TME, such as the ECM pathway (Sangaletti et al., 2017), were enriched by GSEA analysis. The previous study has proven that the invasion and survival of tumor cells can be promoted by ECM-mediated signaling (Conklin and Keely, 2012). Cancer patients, such as pancreatic and colorectal cancer, with high levels of ECM change deposition have a poor prognosis (Levental et al., 2009; Calon et al., 2015; Isella et al., 2015). Similarly, several studies have suggested that abnormal activation of focal adhesion and JAK-STAT was associated with progression and poor prognosis of ovarian cancer (Yang et al., 2019; Zhang J. et al., 2022).

We further observed the different sensitivity to chemotherapy drugs in two risk groups. The risk scores obtained from the WSIs had a low correlation coefficient with the TIDE score, which meant that the risk score was not a good predictor of response to immunotherapy. Our findings demonstrated that the high-risk score group had higher IC50 levels for several chemotherapeutic drugs, indicating the OC patients with low-risk scores were more responsive to the selected drugs. Additionally, the risk score can be utilized as an independent prognostic factor. By combining risk score with age to draw a nomogram, the model had a stable and powerful survival predictive capability. However, some limitations of this research should be noted. Firstly, the risk score has not been validated in external clinical settings, although we are planning the clinical validation of our WSI datasets. Additionally, prospective multi-center studies may be needed to test our model and to overcome possible biases of the retrospective research.

In summary, this study proposed a deep learning framework based on WSI to predict patient prognosis in OC. We believed that the prognostic indicator has the possibility of being used by clinicians to improve decision-making.

## Data Availability

The datasets presented in this study can be found in online repositories. The names of the repository/repositories and accession number(s) can be found in the article/Supplementary Material.
